# Primary B cell Lymphoma of the tongue: a case report

**Published:** 2012-05-06

**Authors:** Mounir Hmidi, Nabil Touiheme, Ali Elboukhari, Mounir Kettani, Charafeddine Elmejareb, Abdelhamid Messary

**Affiliations:** 1 Department Otolaryngology and Neck Surgery, Military Hospital Moulay Ismail, Meknès, Morocco; 2 Department of Pathology, Military Hospital My Ismail Meknès, Morocco

**Keywords:** Lymphoma, non-Hodgkin, tongue, extranodal lymphoma

## Abstract

Malignant lymphoma of the oral cavity is rare and of the tongue even rarer. Location of oral lymphomas is more frequent in masticatory mucosa than in movable mucosa; the lingual and buccal mucosa is rarely involved; whereas the gingival vestibule and Waldeyer's ring seem to be the most frequent site of occurrence. We describe a 78 year old male who presented with a mass lesion primarily involving the base of tongue and was diagnosed as diffuse large B cell lymphoma. The patient was treated with CHOP chemotherapy and radiotherapy. The lesion was completely disappeared). He has currently remained disease free for 16 months. Although oral lymphoma of tongue is very uncommon, it should always be considered in differential diagnosis of various benign and malignant lesions in this region. A proper clinical evaluation, histopathologic as well as immunohistochemical evaluation of biopsy specimen may aid in the diagnosis and thus, help in proper management.

## Introduction

Lymphomas form a group of uncommon solid malignant tumors with a wide spectrum of clinical and pathological features. Primary Non-Hodgkin's Lymphoma (NHL) represents the second leading malignancy of oral cavity, after squamous cell carcinoma [1]. Location of oral lymphomas is more frequent in masticatory mucosa than in movable mucosa; the lingual and buccal mucosa are rarely involved [2], whereas the gingival vestibule and Waldeyer's ring seem to be the most frequent site of occurrence [1].

Most of the few reported cases of primary extranodal non-Hodgkin's lymphoma of the tongue had associated cervical lymph node involvement and manifested as an ulcerated exophytic lesion [3].we report a case of a patient with diffuse large B-cell lymphoma of the base of the tongue, without any superficial ulceration or lymph node involvement.

## Patient and case report

A 78 year old male patient presented with a three month history of dysphagia, shortness of breath and right-sided pain radiating to the ear. No weight loss, night sweats or fever were reported. There was no history of hoarseness. His medical history was unremarkable. No medications were taken on a regular basis, there were no known allergies and the patient was a smoker for 25 years.

Oral examination by observation showed an obvious asymmetry of the tongue base. Digital palpation revealed a hard and large sub-mucosal mass involving the pharyngeal tongue especially in the left side. There was no ulceration or superficial growth on the surface of the tongue. The mobility of the oral tongue was unaffected. The left pharyngeal wall and tonsil were normal and no cervical lymph nodes were clinically apparent. His Systemic examination including respiratory, cardiac, abdominal and central nervous system were normal. Routine investigations: hemogram, urine analysis, chest X-ray and OPG (ortho-pantomogram), Chest radiograph, head, neck were normal. Serology for human immunodeficiency virus was negative.

**Figure 1:**
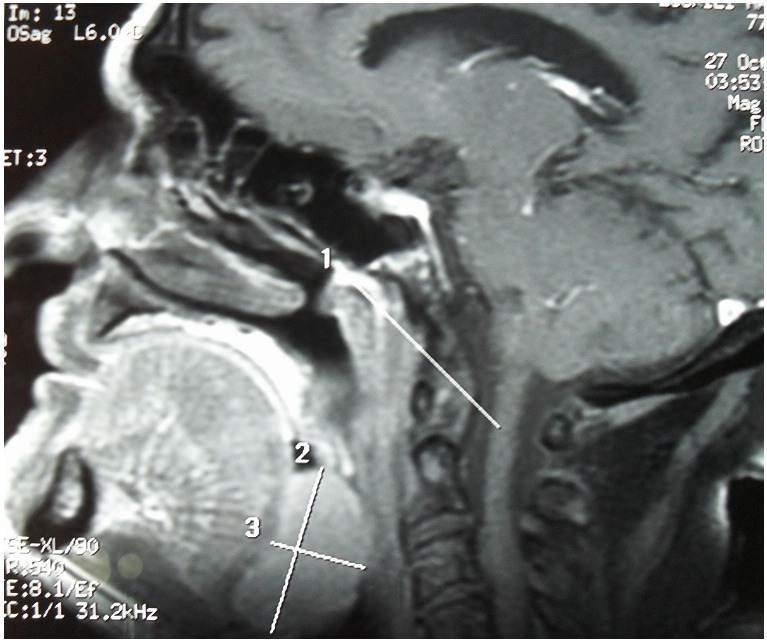
MRI showed a raised mass in the base of the tongue which filled and reduced the light of the oropharynx and the left vallecula

**Figure 2:**
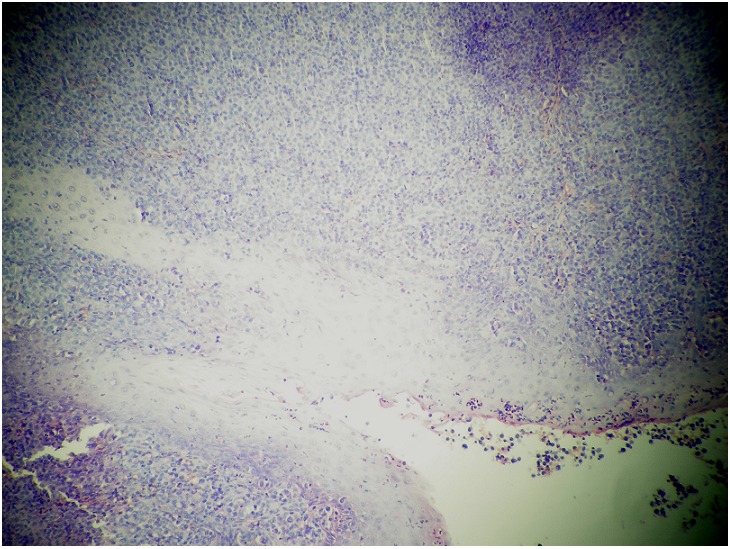
Histological examination shows the presence of diffuse large B cell lymphoma centroblastic variant (HE x 200)

**Figure 3:**
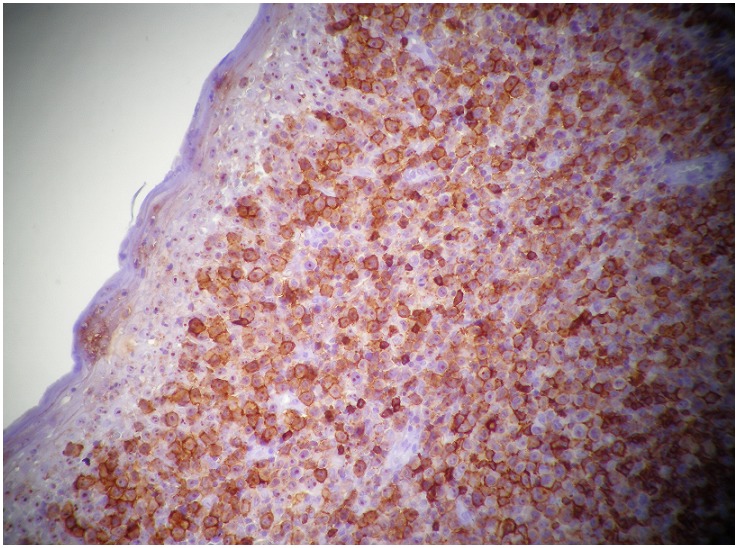
Immunohistochemical testing showed the large tumor cells express CD 20

A Magnetic Resonance Imaging(MRI) revealed a raised mass (3.4cm x 1.6cm x 3.6cm) in the base of the tongue which filled and reduced the light of the oropharynx and the left vallecula (Figure 1). No enlarged cervical lymph nodes were seen. Biopsy of the lesion on histopathological examination demonstrated diffuse infiltration by B cell lymphoma centroblastic variant (Figure 2). A diagnosis of primary B cell lymphoma of the tongue was made according to the immunohistochemical test results showing the lamina propria was infiltrated by a medium sized to large lymphoid cells with oval to round vesicular nuclei, fine chromatin and multiple nucleoli. The cytoplasm was scanty and amphophilic to basophilic (Figure 3). The patient was extensively investigated for other sites of involvement. Bone marrow aspiration, thoracic and abdominal CAT scan was performed. No other sites in the body were found to be affected by the disease.

The patient was treated with chemotherapy which consisted of four courses of CHOP (cyclophosphamide, doxorubicin, vincristine, prednisone). After four cycles of chemotherapy, the lesion was completely disappeared. The treatment was followed by radiotherapy (40Gy). He has currently remained disease free for 16 months.

### Consent

Written informed consent was obtained for publication of this case report and accompanying images.

## Discussion

Malignant lymphoma of the oral cavity is rare [3], 20 to 30% of non-Hodgkin's lymphoma arise from extranodal sites [4]. The head and neck is the second most common region for extranodal lymphoma after the gastrointestinal tract [4].

Primary malignant lymphoma a of the tongue is rare; we could find only eight cases of primary tongue lymphoma reported in English [4, 5]. It generally affects the elderly, especially over the 6th decade of life [4], and the male: female ratio was 6:3 [4, 5]. There are no characteristic clinical features of non-Hodgkin's lymphoma of the oral region. The most common presenting symptoms are local swelling, pain or discomfort and ulcer. The tumor may manifest as a submucosal mass, a polypoid bulky mass with a smooth mucosal surface, or as an ulcerated lesion. Involvement of the intrinsic tongue musculature causes restriction of movement, dysarthria and dysphagia [4]. Occasionally, the tumor may cause upper airway obstruction which is the case of our patient. CT and MRI were diagnostic in most cases; the diagnosis was confirmed by incisional biopsy and immunohistochemical studies in all cases [5]. Differential diagnosis includes metastatic tumors in the tongue, melanomas, poorly differentiated squamous cell carcinomas, poorly differentiated adenocarcinomas, and rare tumors such as neuroblastomas, rhabdomyosarcomas and Ewing's tumor [6].

From the small number of well documented case reports of primary extranodal non-Hodgkin's lymphoma of the tongue, little is known about the etiological factors for primary lymphoma of the oral region. Few cases have been reported in which the presenting symptom was a submucosal mass with no exophytic lesion or ulceration [6, 7]. Of these, only two cases presenting without cervical lymph node involvement have been described [7, 8]. In addition, little cases were associated with Acquired Immune Deficiency Syndrome (AIDS) [4]. we have presented a rare case of extranodal primary non-Hodgkin's lymphoma of the pharyngeal tongue. This case is unusual in that there was only submucosal involvement detected by palpation and no associated cervical lymph node involvement was seen. We stress the importance of digital palpation of the tongue base in patients complaining of dysphagia or a foreign body sensation in the throat.

## Conclusion

Although oral lymphoma of tongue is very uncommon, it should always be considered in differential diagnosis of various benign and malignant lesions in this region. A proper clinical evaluation, histopathologic as well as immunohistochemical evaluation of biopsy specimen may aid in the diagnosis and thus, help in proper management.
